# Temporal and spectral EEG dynamics can be indicators of stealth placement

**DOI:** 10.1038/s41598-018-27294-4

**Published:** 2018-06-14

**Authors:** Regina W. Y. Wang, Yi-Chung Chen, I-Ning Liu, Shang-Wen Chuang

**Affiliations:** 1 Design Perceptual Awareness Lab (D:PAL), National Taiwan University of Science and Technology (Taiwan Tech), Taipei, Taiwan; 2 The Department of Industrial and Commercial Design, National Taiwan University of Science and Technology (Taiwan Tech), Taipei, Taiwan; 3 Taiwan Building Technology Center, National Taiwan University of Science and Technology (Taiwan Tech), Taipei, Taiwan

## Abstract

Stealth placement marketing, where consumers are unaware that they are being marketed to, attempts to reduce the audiences’ resistance to traditional persuasive advertising. It is a form of advertising that involves targeted exposure of brands or products incorporated in other works, usually with or without explicit reference to the brands or products. Brand placement can be presented in different visual and auditory forms in video programs. The present study proposed that different ‘representations’ (i.e., representable or non-representable) and ‘sounds’ (i.e., speech or musical sound) of brand placement can affect the viewers’ perception of the brand. Event-related potential results indicated significant differences in P1, N1, P2, N270, and P3. Further, event-related spectral perturbation results indicated significant differences in theta, alpha, beta, and gamma (30–100 Hz), in the right parietal, right occipital area, and limbic lobe. ‘Non-representable’ or ‘speech sound’ brand placement induced significant temporal and spectral EEG dynamics in viewers.

## Introduction

Electroencephalography^1^ has been frequently by academics and industries to analyse the effects of advertisements and placement marketing^2–6^. EEG can record the state of the participant’s brain activity when continuously viewing videos, while highly accurate temporal and spectral EEG dynamics can be used to examine the participant’s immediate internal responses^3,7^. This facilitates the precise analysis of consumers’ perception of stealth brand placement. Processing the consumers’ perception when they see a branded product during a movie, television show, or video is crucial. Studies have demonstrated that early spatial attention and discrimination enhances the P1 in the occipital areas^8–10^. P2 is related to early auditory discrimination^11^. P1 and P2 are related to the effects under combined visual and auditory stimulation^12,13^. N1 has been considered a signal for the allocation of attentional resources^14^. Late components, i.e., P3 or LPC, are related to the processing of stimuli-induced emotions and preferences^15–17^, and can be used to detect marketing effects in research^6,18^. The alpha band in the posterior parietal cortex (PPC) is related to spatial attentional shift of different sensory modes^19^, whereas the alpha and beta bands in the occipital region are related to preparatory attention^20^. When viewing advertisements, the activation level of the theta band is the basis for judging pleasure^5^. Gamma activation is related to conscious awareness and selective attention^21,22^. Conscious awareness evokes the gamma band in the visual cortex, while spatial attention evokes higher frequencies in the gamma range^21^. Studies have also shown that the brain processes music and speech in different hemispheres of the auditory cortex^23–25^, particularly the temporal lobe, while the left hemisphere analyses speech^26^ and right hemisphere analyses music.

Rance Crain, the editor-in-chief of *Advertising Age*, once said that ‘Advertisers will not be satisfied until they put their brand mark on every blade of grass’^27^. Thus, studies, including the present one, can determine whether brand placement anywhere, at any time, is a popular type of advertising. However, the crude and excessive marketing placement could annoy the audience. For example, in 2011, 71 brands were placed in the film *Transformers: Dark of the Moon*, which caused a backlash among the audience^28^. In the study by d’Astous and Seguin^29^, brand placement in the background that was unrelated to the program content led to the most negative evaluation. The present study examined the categorization of branded products according to their benefits and relevance to a program as follows: (a) ‘Non-integrated brand placement’ involves the direct inclusion of the brand logo and products before, during, or after the program, but where the program content is not associated with the benefits of the branded product^30,31^. For example, alcoholic beverage manufacturers may sponsor singing programs, which despite the irrelevance of its logo or products to singing, can still be included in the program; (b) ‘Integrated brand placement’ involves the inclusion of brand logos and products in a plot-relevant manner in the program. For example, in the 007 film series, the main character always drives famous cars when pursuing the enemy, which not only highlights the performance of the car but is also linked to the content of the film. The visual and auditory performances of TV programs could impact the cognition and preferences of the viewers^32–35^. Brand placement can be presented in different visual and auditory forms in programs. Our main topic of interest is the audience’s attitude and cognitive processes towards the brands.

Stealth, product, or brand placement or embedded advertisement is a subtler form of advertising technique involving the exposure of brands or products incorporated into another work in different existing medias^36–38^. Most placements are commonly featured in movies, television shows, and videos, usually with or without explicit reference to the brands or products. Advertising and marketing involves the attainment of various communication goals and product sales by targeting the consumers’ demands^39^. The stages of consumer response to advertising communication have been previously summarised by a number of scholars^40–42^. This process specifically involves the ‘receipt of advertising information’, ‘idea generation’, and ‘purchasing decision’. The ‘receipt of advertising information’ stage is the process where the viewers perceive and recognise the advertising. The ‘idea generation’ stage involves the liking, preferences, and attitudes generated by the viewers following exposure to the advertisement. The ‘purchasing decision’ stage is where the viewers form their purchase intention towards the branded product following the generation of brand-related ideas. This demonstrates that the viewers’ cognition and perception begin when they attend to the brand. Visual awareness is the post-attentional behaviour of producing consciousness^21^. Further, consumers can only produce preference decision and purchasing desire towards brands after the generation of awareness. Therefore, the present study examined cognitive processes for ‘representation’ and ‘sound’ by using the consumers’ awareness, preferences, and purchasing desire towards brand placement. In the present study, the representation of brand placement is divided into representable brand placement (REBPL) and non-representable brand placement (NREBPL). REBPL is the presentation of brand logos and products within the actual scenes of the video They appear naturally and randomly at different locations within the scenes according to the needs of the program script and have reasonable correlation with the development of the script content. NREBPL is the graphic processing of brand logo and product information, embedding the brand into the same location of each video frame and permeating the entire program, without reasonable correlation with the development of the program script.

Sound is an important element in film^33,43,44^. Speech and music are the two main types of sounds in program recording^32,43^. Sounds can often alter and stimulate people’s emotions^45,46^. Speech and music differ in their acoustic properties and rhythm^40,47^. The vibrations of any sound-producing apparatus can produce sound. Speech is formed from several sound waves that are produced within a period by one type of sound-producing apparatus (e.g., human vocal cords). Within this period, the frequency changes of vocal sound waves are large and unstable. The current study defined speech sound brand placement (SSBPL) as the presentation of brand logo and product information in the program together with speech sounds reading the brand name aloud. Music is formed of several sound waves produced within a period by more than one type of sound-producing apparatus (including human vocal cords). Within this period, the frequency changes of music sound waves are small and relatively stable. Moreover, musical sound brand placement (MSBPL) was defined in the current study as the presentation of brand logo and product information with music.

**Figure 1:**
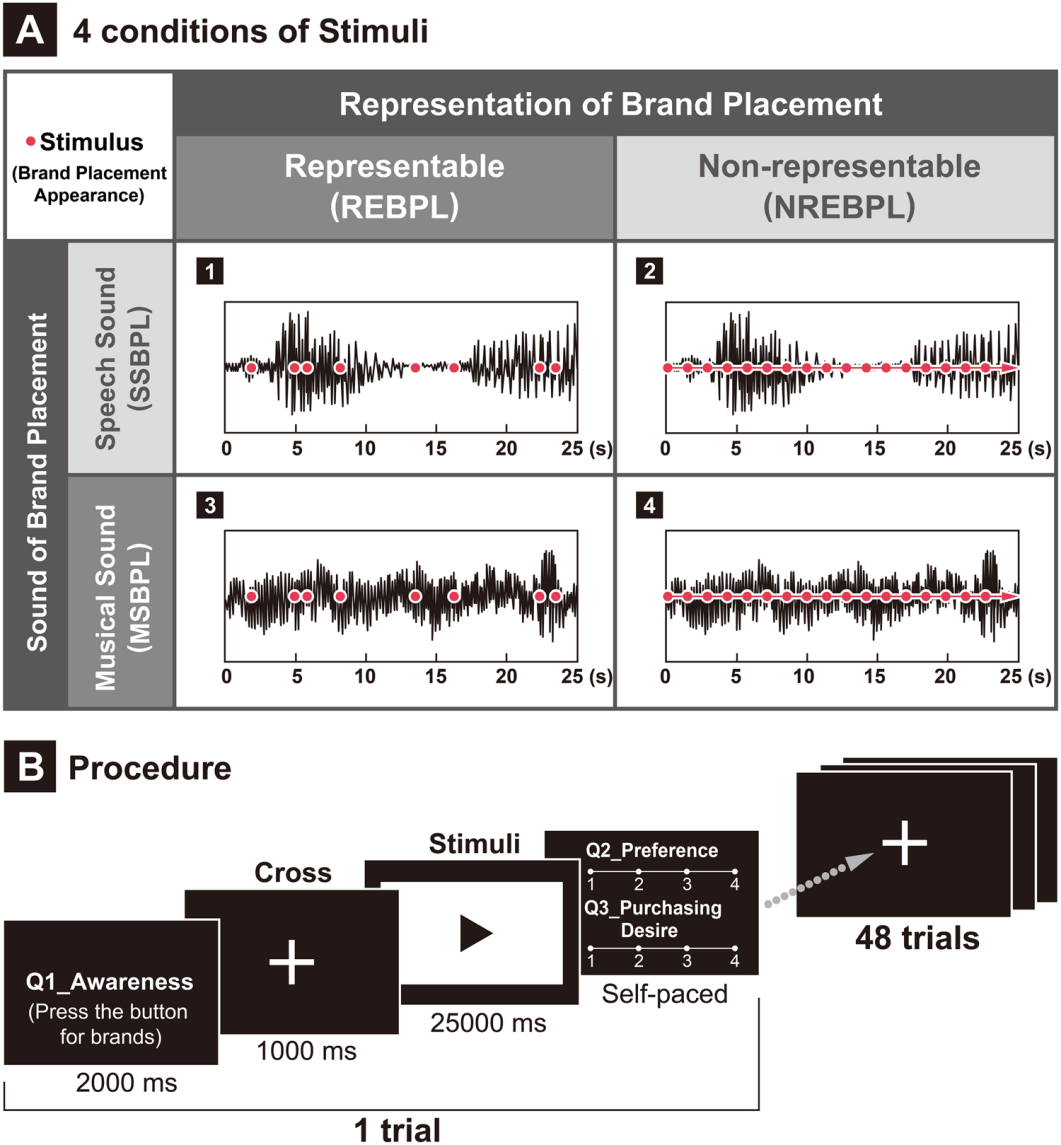
(**A**) Design of the experimental stimuli. A 2 × 2 framework was used to examine the effects of the interaction between representable/non-representable and speech/musical sounds on brand placement. The 4 types of stimulus condition were as follows: (1) ‘representable versus speech sound’ brand placement, (2) ‘non-representable versus speech sound’ brand placement, (3) ‘representable versus musical sound’ brand placement, and (4) ‘non-representable versus musical sound’ brand placement. (**B**) The experimental procedure first involved the presentation of a behavioural cue before the stimulus video, followed by a ‘+’ fixation point for 1,000 ms and a stimulus video for 25,000 ms. The behavioural questions were then presented; the participants controlled the timing. The experimental process for one stimulus video is presented here. The entire experiment, which included 48 trials and 105 stimuli, lasted for approximately 35 min.

The present study used the representation or sound of brand placement as independent variables to test the effects of brand placement on the viewers’ discrimination and preferences, with reference to brain activity indicators. The hypotheses of our study were as follows: (1) More representable brand placement will have greater effects on the temporal and spectral EEG dynamics of viewers’ discrimination and preferences; (2) brand placement with more music accompaniment will have greater effects on the temporal and spectral EEG dynamics of the discrimination and preferences of viewers; and (3) more representable brand placement with more music accompaniment will have greater interaction effects on the temporal and spectral EEG dynamics of the viewers’ discrimination and preferences. The stimuli and experimental design are shown in Fig. 1.

## Results

### Behavioural Results

**Figure 2:**
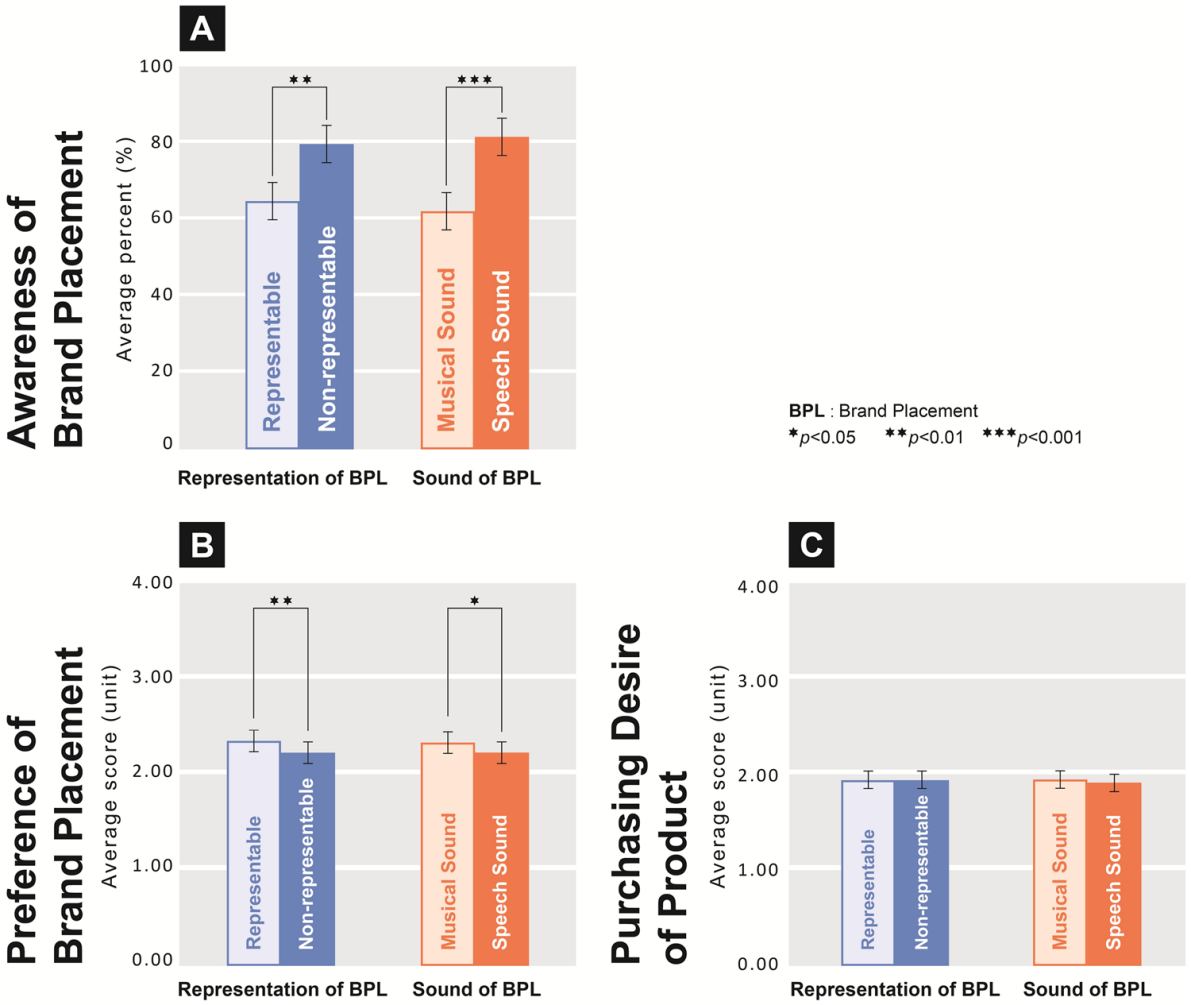
Behavioural results indicated that the two factors of brand placement led to significant differences in awareness, preference, and purchasing desire. (**A**) Under the NREBPL condition, awareness of SSBPL was significantly higher than that of MSBPL (*p* < 0.001). (**B**) Under the SSBPL condition, awareness of NREBPL was significantly higher than that of REBPL (*p* < 0.001). (**C**) Preference of the REBPL condition was significantly higher than that of NREBPL (*p* < 0.001); preference of the MSBPL condition was significantly higher than that of SSBPL (*p* < 0.05). (**D**) There was no significant difference in purchasing desire between the REBPL and NREBPL conditions (*p* > 0.05), and between the MSBPL and SSBPL conditions (*p* > 0.05).

The behavioural results and statistical data (Fig. 2 and Table 1) indicate the effects of the 4 stimulus conditions on the participants’ ‘awareness’, ‘preference’, and ‘purchasing desire’. The results indicated that the main effect of representation was significant (*F* = 10.795, *df* = 1, *p* = 0.001). Further, the awareness of NREBPL (*M* = 0.792, SD = 0.219) was significantly higher than that of REBPL (*M* = 0.651, SD = 0.212). The main effect of sound was significant (*F* = 26.524, *df* = 1, *p* < 0.001); the awareness of SSBPL (*M* = 0.825, SD = 0.206) was significantly higher than that of MSBPL (*M* = 0.618, SD = 0.198; Fig. 2A).

**Table 1:** One-way analysis of variance for different representations and sounds in 3 variables.

Source of variation	SS	*df*	MS	*F*	*p*	Main Effect
**Awareness of Brand Placement**						
Representation	1.073	1	0.500	10.795	**0**.001**	NREBPL > REBPL
Sound	0.500	1	1.073	26.524	**0**.000***	SSBPL > MSBPL
**Preference of Brand Placement**						
Representation	4.083	1	4.083	7.346	**0**.007**	REBPL > NREBPL
Sound	2.430	1	2.430	4.361	**0**.037*	MSBPL > SSBPL
**Purchasing Desire of Product**						
Representation	0.003	1	0.003	0.007	0.934	—
Sound	0.480	1	0.480	0.979	0.323	—

***p < 0.001, **p < 0.01, *p < 0.05; SS: sum of squares of deviation from mean; df: degree of freedom; MS: mean square

A one-way analysis of variance was performed on the representation and sound data to analyse the participants’ ‘preference’ and ‘purchasing desire’ of brand placement. The results indicated that the main effect of representation was significant (*F* = 7.346, *df* = 1, *p* = 0.007 or <0.01); the preference of REBPL (*M* = 2.310, SD = 0.763) was significantly higher than that of NREBPL (*M* = 2.193, SD = 0.728). Further, the main effect of sound was significant (*F* = 4.361, *df* = 1, *p* = 0.037 or <0.05); the preference of MSBPL (*M* = 2.297, SD = 0.725) was significantly higher than that of SSBPL (*M* = 2.207, SD = 0.767; Fig. 2B). The main effects of representation (*F* = 0.007, *df* = 1, *p* = 0.934 or >0.05) and sound (*F* = 0.979, *df* = 1, *p* = 0.323 or >0.05), however, were both not significant (Fig. 2C).

### Event-Related Potential Results

**Figure 3:**
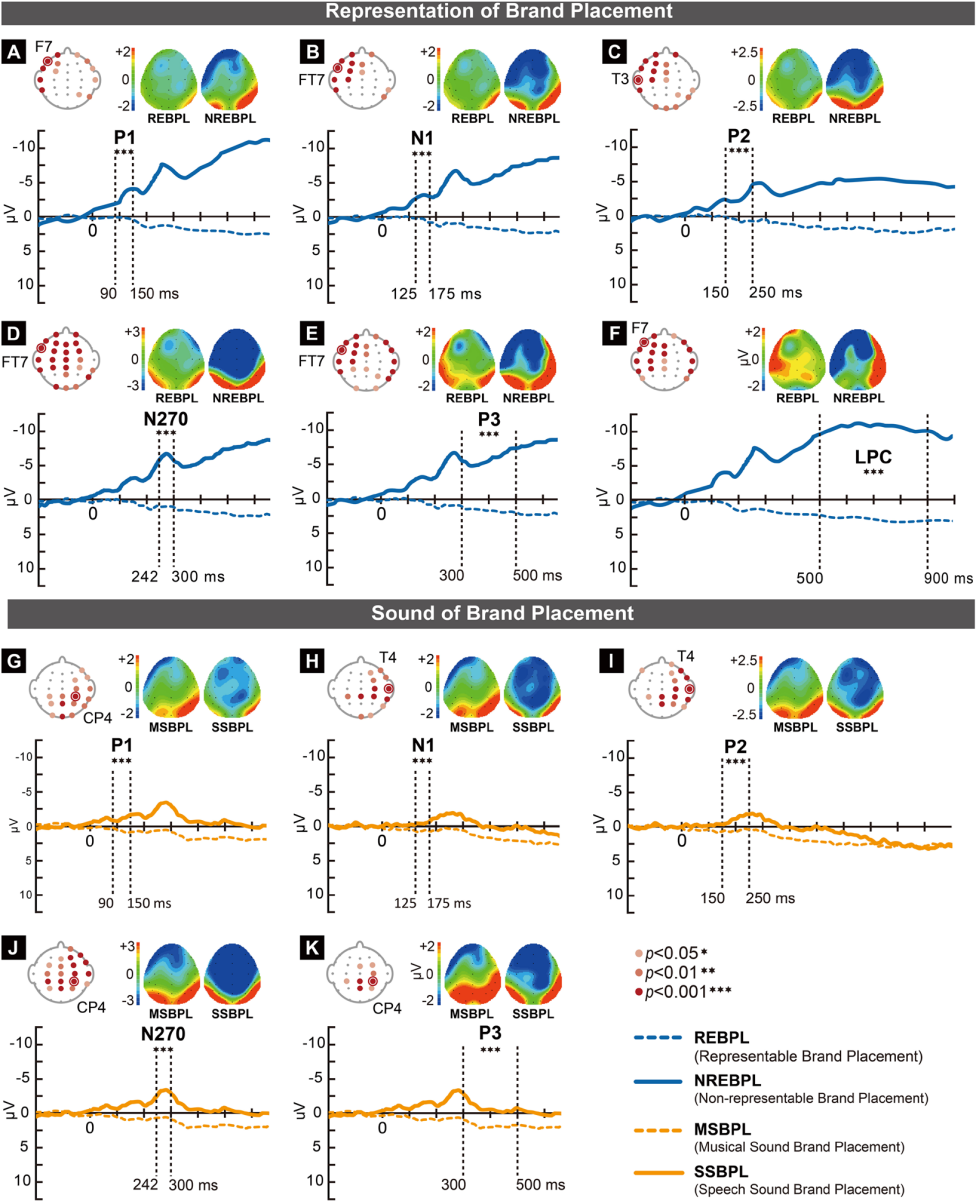
(**A**–**K**) Significant electrode maps, brain heat maps, and maximum average amplitude of event-related potential (ERP) components with significant differences in representation and sound of brand placement. ☉ indicates significant electrodes in the ERP waveform (*p* < 0.001). (**A**–**F**) show the significant electrode maps of P1, N1, P2, N270, P3, and LPC, with significant differences in the different representations of brand placement. (**A**) For the ERP component P1 at electrode F7, the maximum average amplitude of REBPL was higher than that of NREBPL (*p* < 0.001). (**B**) For the ERP component P1 at electrode FT7, the maximum average amplitude of NREBPL was higher than that of REBPL (*p* < 0.001). (**C**) For the ERP component P2 at electrode T3, the maximum average amplitude of REBPL was higher than that of NREBPL (*p* < 0.001). (**D**) For the ERP component N270 at electrode FT7, the maximum average amplitude of NREBPL was higher than that of REBPL (*p* < 0.001). (**E**) For the ERP component P3 at electrode FT7, the maximum average amplitude of REBPL was higher than that of NREBPL (*p* < 0.001). (**F**) For the ERP component LPC at electrode F7, the maximum average amplitude of REBPL was higher than that of NREBPL (*p* < 0.001). (**G**–**K**) show significant electrode maps for P1, N1, P2, N270, and P3, with significant differences for different sounds of brand placement. (**G**) For the ERP component P1 at electrode CP4, the maximum average amplitude of MSBPL was higher than that of SSBPL (*p* < 0.001). (**H**) For the ERP component N1 at electrode T4, the maximum average amplitude of SSBPL was higher than that of MSBPL (*p* < 0.001). (**I**) For the ERP component P2 at electrode T4, the maximum average amplitude of MSBPL was higher than that of SSBPL (*p* < 0.001). (**J**) For the ERP component N270 at electrode CP4, the maximum average amplitude of SSBPL was higher than that of MSBPL (*p* < 0.001). (**K**) For the ERP component P3 at electrode CP4, the maximum average amplitude of MSBPL was higher than that of SSBPL (*p* < 0.001).

The ERP analysis involved a comparison of the maximum average amplitudes between the two levels of a single independent variable within a period. Manipulation of the two independent variables of brand placement in the present study demonstrated significant differences between the different representations (*p* < 0.05) at P1 (90–150 ms)^8,48^, N1 (125–175 ms)^49–51^, P2 (150–250 ms)^13,51,52^, N270 (242–300 ms)^53^, P3 (300–500 ms)^16,54^, and LPC (500–900 ms)^16^. Further, different sounds showed significant differences at P1, N1, P2, N270, and P3 (*p* < 0.05; Fig. 3).

Independent-samples *T* tests were performed on the average ERP component values of each electrode for the different representations, i.e., REBPL and NREBPL. The results indicated that differences in the significant electrodes of P1 occurred at the frontal, temporal, and parietal areas (*n* = 13, *p* < 0.05). The average amplitudes of the significant electrodes at FP1, FP2, F7, F3, FT7, FC3, T3, and TP7 were higher for REBPL than for NREBPL (*p* < 0.05), and those at F8, FT8, T4, T6, and P4 were higher for NREBPL than for REBPL (*p* < 0.05). Differences in the significant electrodes of N1 occurred at the frontal and temporal areas (*n* = 13, *p* < 0.05). The average amplitudes of the significant electrodes at FP1, FP2, F7, FZ, F3, FT7, FC3, FCZ, T3, C3, and TP7 were higher for REBPL than for NREBPL (*p* < 0.05). Further, those for the average amplitudes of the significant electrodes at TP8 and T6 were higher for NREBPL than for REBPL (*p* < 0.05). Differences in the significant electrodes of P2 occurred at the frontal, temporal, and parietal areas (*n* = 18, *p* < 0.05). The average amplitudes of the significant electrodes at FP1, FP2, F7, F3, FZ, FT7, FC3, FCZ, T3, C3, CZ, and TP7 were higher for REBPL than for NREBPL (*p* < 0.05), and those at TP8, T6, P4, O1, OZ, and O2 were higher for NREBPL than for REBPL (*p* < 0.05). Differences in the significant electrodes of N270 occurred at the frontal, temporal, central, and parietal areas (*n* = 24, *p* < 0.05). The average amplitudes of the significant electrodes at FP1, FP2, F7, F3, FZ, F4, FT7, FC3, FCZ, FC4, T3, C3, CZ, C4, TP7, CP3, CPZ, CP4, and PZ were higher for REBPL than for NREBPL (*p* < 0.05), and those at TP8, T6, O1, OZ, and O2 were higher for NREBPL than for REBPL (*p* < 0.05). Differences in the significant electrodes of P3 occurred at the frontal, temporal, and parietal areas (*n* = 21, *p* < 0.05). The average amplitudes of the significant electrodes at FP1, FP2, F7, F3, FZ, FT7, FC3, FCZ, T3, C3, TP7, CP3, and CPZ were higher for REBPL than for NREBPL (*p* < 0.05), while those at F8, FT8, T4, TP8, T6, P4, O1, and O2 were higher for NREBPL than for REBPL (*p* < 0.05). Differences in the significant electrodes of LPC occurred at the frontal, temporal, central, and parietal areas (*n* = 20, *p* < 0.05). The average amplitudes of the significant electrodes at FP1, FP2, F7, F3, FZ, FT7, FC3, FCZ, T3, C3, CZ, TP7, T5, CP3, and CPZ were higher for REBPL than for NREBPL (*p* < 0.05), and those at F8, FT8, T4, T6, and TP8 were higher for NREBPL than for REBPL (*p* < 0.05).

Independent-samples *T* tests were performed on the average ERP component values of each electrode for different sounds, i.e., SSBPL and MSBPL. There were significant differences between the P1 electrodes at the frontal, temporal, parietal, and occipital areas (*n* = 13, *p* < 0.05). The average amplitudes of the significant electrodes at F8, FC4, FT8, C4, T4, CP3, CPZ, CP4, TP8, T6, P3, PZ, P4, O1, OZ, and O2 were higher for MSBPL than for SSBPL (*p* < 0.05). Significant differences between N1 electrodes occurred at the frontal, temporal, parietal, and occipital areas (*n* = 13, *p* < 0.05). The average amplitudes of the significant electrodes at F4, F8, FC4, FT8, C4, T4, CP3, CPZ, CP4, TP8, T6, OZ, and O2 were higher for SSBPL than for MSBPL (*p* < 0.05). Differences between P2 electrodes were significant at the frontal, temporal, and parietal areas (*n* = 16, *p* < 0.05). The average amplitudes of the significant electrodes at FP2, F7, F4, F8, FC4, C4, T4, TP3, TPZ, TP4, TP8, PZ, P4, and T6 were higher for MSBPL than for SSBPL (*p* < 0.05). Significant differences between N270 electrodes were present at the frontal, temporal, and parietal areas (*n* = 19, *p* < 0.05). The average amplitudes of the significant electrodes at FP2, FZ, F4, F8, FC3, FCZ, FC4, FT8, C3, CZ, C4, T4, CP3, CPZ, CP4, TP8, C3, CZ, and C were higher for SSBPL than for MSBPL (*p* < 0.05). Differences between P3 electrodes occurred at the frontocentral and parietal areas (*n* = 8, *p* < 0.05). The average amplitudes of the significant electrodes at FCZ, FC4, C4, CP3, CPZ, CP4, PZ, and P4 were higher for MSBPL than for SSBPL (*p* < 0.05).

### Event-related spectral perturbation Results

**Figure 4:**
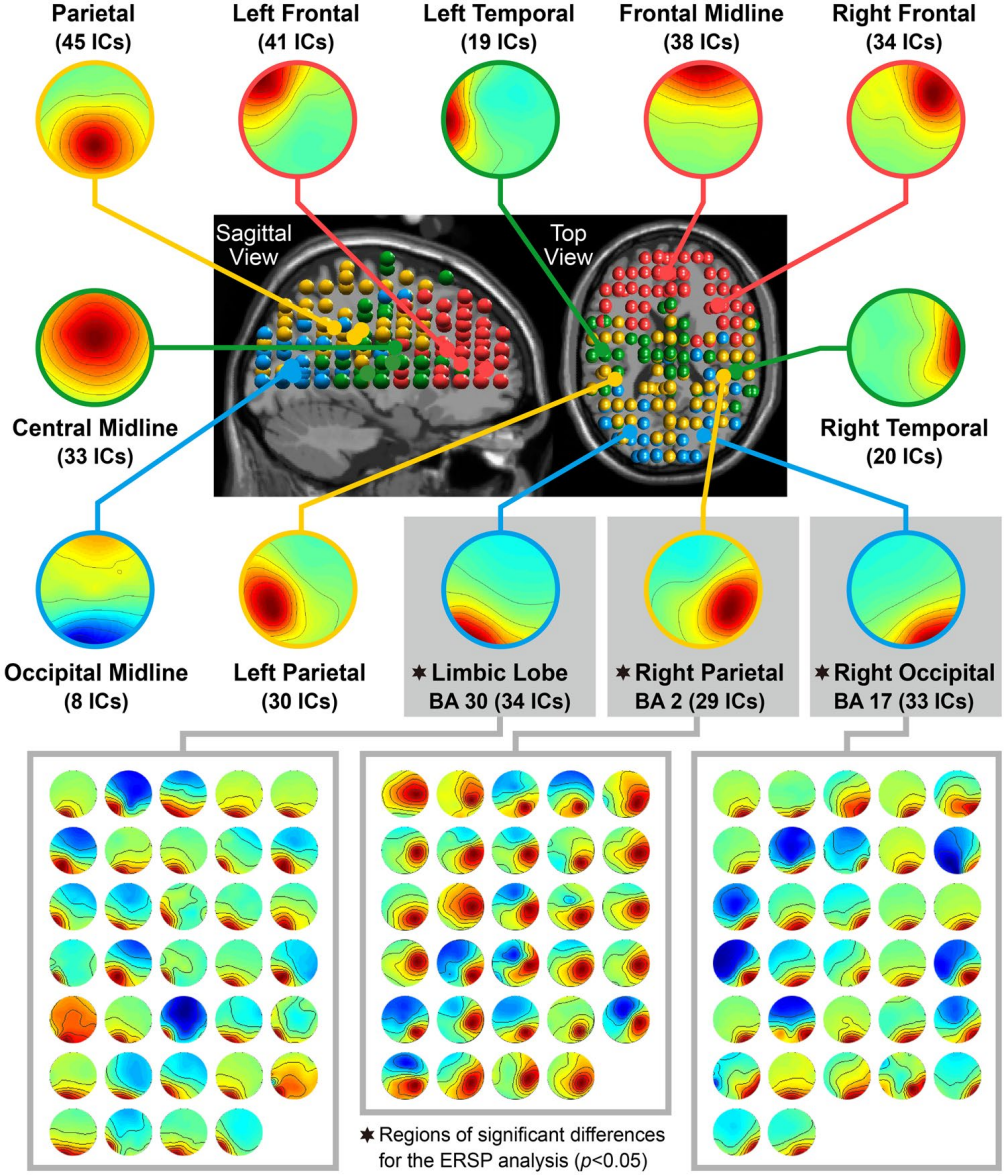
Independent component analysis was performed on the electroencephalography signals of all the participants to obtain the statistically significant scalp map and equivalent dipole for clustering, which resulted in 12 brain areas. The figures show the scalp map and equivalent dipole of the 12 brain areas. *Indicates that the ERSP analysis revealed significant differences in these brain regions for the two factors (different representations and sounds of brand placement; *p* < 0.05), which include the right parietal area (BA 2), right occipital area (BA 17), and limbic lobe (BA 30). Parametric testing with FDR correction was performed for significance testing (*p* < 0.05) to further compare the differences for the varying representations and sounds at different frequencies (Fig. 5).

Independent component analysis (ICA) of the EEG signals from all the participants was performed to obtain the locations of significant brain regions, equivalent dipole locations, and a scalp map (Fig. 4). The event-related spectral perturbation (ERSP) analysis revealed that different representations and sounds of brand placement led to significant differences in the right parietal area (BA 2), right occipital area (BA 17), and limbic lobe (BA 30; *p* < 0.05).

**Figure 5:**
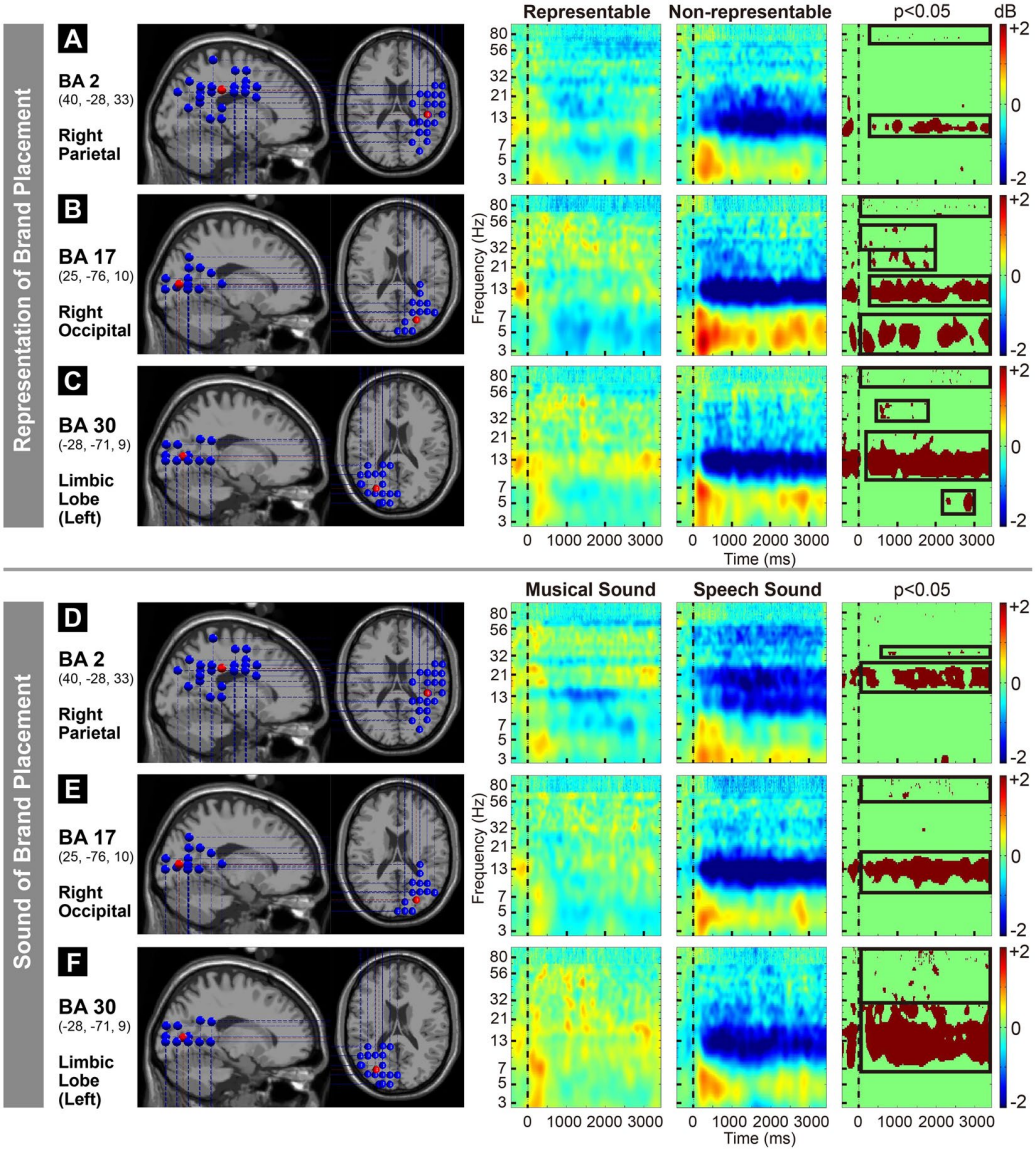
Event-related spectral perturbation (ERSP) results of the 25 participants for different brand placement representations and sounds. False discovery rate analysis was performed for significance testing. (**A**–**C**) Brain regions and ERSP(s) results are shown, with significant differences between REBPL and NREBPL. The two factors indicate significant differences in the spectral powers of theta, alpha, beta, and gamma for Brodmann areas BA 2, BA 17, and BA 30 (*p* < 0.05). (**A**) In BA 2, REBPL shows a higher spectral power for 7–13 Hz (alpha) than NREBPL (*p* < 0.05), and NREBPL shows a higher spectral power for 80–100 Hz (high gamma) than REBPL (*p* < 0.05). (**B**) In BA 17, NREBPL shows a higher spectral power for 3–6 Hz (theta) than REBPL (*p* < 0.05); REBPL shows a higher spectral power for 9–20 Hz (alpha, beta) than NREBPL (*p* < 0.05); REBPL shows a higher spectral power for 20–30 Hz (beta) than NREBPL (*p* < 0.05); REBPL shows a higher spectral power for 30–60 Hz (low gamma) than NREBPL (*p* < 0.05); and NREBPL shows a higher spectral power for 60–100 Hz (high gamma) than REBPL (*p* < 0.05). (**C**) In BA 30, NREBPL shows a higher spectral power for 4–6 Hz (theta) than REBPL (*p* < 0.05); REBPL shows a higher spectral power for 9–20 Hz (alpha, beta) than NREBPL (*p* < 0.05); REBPL shows a higher spectral power for 30–40 Hz (low gamma) than NREBPL (*p* < 0.05); and NREBPL shows a higher spectral power for 60–100 Hz (high gamma) than REBPL (*p* < 0.05). (**D**–**F**) Brain regions and ERSP(s) results are shown, with significant differences between SSBPL and MSBPL. The two factors indicate significant differences in the spectral powers of alpha, beta, and gamma for Brodmann areas BA 2, BA 17, and BA 30 (*p* < 0.05). (**D**) In BA 2, MSBPL shows a higher spectral power for 15–15 Hz (beta) than SSBPL (*p* < 0.05), and MSBPL shows a higher spectral power for 30–40 Hz (low gamma) than SSBPL (*p* < 0.05). (**E**) In BA 17, MSBPL shows a higher spectral power for 10–20 Hz (alpha, beta) than SSBPL (*p* < 0.05), and MSBPL shows a higher spectral power for 60–100 Hz (high gamma) than SSBPL (*p* < 0.05). (**F**) In BA 30, MSBPL shows a higher spectral power for 7–30 Hz (alpha, beta) than SSBPL (*p* < 0.05), and MSBPL shows a higher spectral power for 30–100 Hz (low gamma, high gamma) than SSBPL (*p* < 0.05).

Figure 5A shows the significant differences in 9–13 Hz (alpha; *p* < 0.05) and 80–100 Hz (high gamma) between REBPL and NREBPL in the right parietal area (BA 2). Figure 5B shows significant differences in 3–6 Hz (theta; *p* < 0.05), 9–20 Hz (alpha, beta; *p* < 0.05), 20–30 Hz (beta; *p* < 0.05), 30–60 Hz (low gamma), and 60–100 Hz (high gamma; *p* < 0.05) between REBPL and NREBPL in the right occipital area (BA 17). Figure 5C shows significant differences in 4–6 Hz (theta; *p* < 0.05), 9–20 Hz (alpha, beta; *p* < 0.05), 30–60 Hz (low gamma), and 60–100 Hz (high gamma; *p* < 0.05) between REBPL and NREBPL in the limbic lobe (BA 30). Figure 5D shows significant differences in 15–20 Hz (beta; *p* < 0.05) and 30–40 Hz (low gamma; *p* < 0.05) between SSBPL and MSBPL in the right parietal area (BA 2). Figure 5E shows significant differences in 10–20 Hz (alpha, beta; *p* < 0.05), and 60–100 Hz (high gamma; *p* < 0.05) between SSBPL and MSBPL in the right occipital area (BA 17). Figure 5F shows significant differences in 7–30 Hz (alpha, beta; *p* < 0.05) and 30–100 Hz (gamma; *p* < 0.05) between SSBPL and MSBPL in the limbic lobe (BA 30).

## Discussion

The experimental hypotheses of the present study were centred on the effects of representable or non-representable product placement, and of speech sound or musical sound brand placements on brain EEG activity. The attention and awareness of non-representable and speech sound brand placements of the viewers were analysed based on the behavioural, ERP, and ERSP data.

Analyses of the behavioural data indicated that the representation and sound of brand placement had a main effect on the viewers’ awareness (see Section 3.1). The viewers’ awareness for NREBPL was higher than for REBPL. Thus, blatant placement led to better viewer recognition than subtle placement^54^. The viewers’ awareness for SSBPL was higher than that for MSBPL. Compared with the recognition of program content using visual information, the use of auditory information under the program was easier^55^, implying that using auditory information alone to determine the video content will allow viewers to avoid the more complicated steps of visual processing^23^.

The present study demonstrated that the representation and sound of brand placement had a main effect on the viewers’ preference. The viewers’ preference was higher for REBPL than for NREBPL, and higher for MSBPL than for SSBPL. Thus, performance-integrated and creative placements are more likely to achieve higher viewer preference and more-positive attitudes^55–57^. Repeated, blatant brand placements will reduce the original impact of the program and lead to negative attitudes in viewers^57^. Background music is a clue to the persuasiveness of advertisements^58^. Using contemporary songs or music can also enhance the viewers’ preference for advertisements^59^.

Moreover, the present findings that the representation and sound of brand placement did not have an interaction or main effect on the viewers’ purchasing desire. Thus, although brand placement in films may enhance brand awareness, it does not seem to have a substantial impact on the viewers’ purchasing desire^60,61^.

The present study demonstrated that more representable brand placement had greater effects on viewers’ temporal and spectral EEG dynamics. Figure 3A–E indicate significant differences between REBPL and NREBPL for P1, N1, P2, N270, P3, and LPC (see Section 3.2). Our results showed that the amplitude of P1 was greater for REBPL than for NREBPL. P1 is evoked at the early stages of visuospatial information processing^10,49,62,63^, while REBPL involves the appearance of brand logo information in real space. P1 also reflects the attentional demands of task processing, such as using brightness or colour to judge the stimulus^10,16^. In contrast, NREBPL uses graphic processing of brand information, such as accentuating and highlighting the brand logo. The N1 amplitude of NREBPL was greater than that of REBPL. N1 is considered the signal for the allocation of attentional resources^14^ and is related to the identification of objects or faces^9^. P2 is related to emotional evaluation^51^; emotionally stimulating words and images evoke larger P2 amplitudes^51,52^. When perceiving emotionally stimulating music or images, the P2 amplitude of positive stimulations is larger than that of negative stimulations^13^. Rare targets can also evoke a larger P2^64^. In the present study, the P2 amplitude evoked by REBPL was larger than that evoked by NREBPL. This might have been because, compared with NREBPL, where the brand is placed in a fixed position in the frame for the entire duration, REBPL involves the natural and random appearance of the brand, which evokes a greater emotional reaction. Moreover, the present findings demonstrated that the N270 amplitude in the frontal area for NREBPL was larger than that for REBPL. The presence of conflict or spatial differences evoke the N270 component^65,66^. N270 has also been used to detect the congruence of brand image and extension products^67^, particularly for frontal lobe activity^53^. In the NREBPL, the viewer may be unable to match the graphic brand logo information occurring throughout the video with the script content of the actual program, leading to a greater sense of conflict. The present study demonstrated that the P3 amplitude in the left frontal and parietal areas for REBPL was larger than that for NREBPL. Visual search tasks will evoke P3 in the frontal lobe^68^, which is an indicator of attentional control mechanisms. Clear selection of attentional targets will evoke a larger P3^48^, while novel and unpredictable stimuli also evokes P3 in the frontal and temporal lobes^69^. Moreover, P3 is an indicator of emotions^16,48^. Further, LPC is a signal of high-level categorization and decisions^70^, with incongruity evoking larger LPC amplitude^71,72^. Our study demonstrated that the LPC amplitudes of the left frontal and temporal areas for REBPL were larger than those for NREBPL. The susceptibility of viewers to the boredom of repeated exposure to visual stimuli will lead to delayed LPC in the frontal lobe^73^.

The present study demonstrated that brand placement accompanied by music was more likely to influence brain activity in the viewers. Figure 3G–K show significant differences between SSBPL and MSBPL in the P1, N1, P2, N270, and P3 amplitudes (see Section 3.2). The P1 amplitude of MSBPL was larger than that of SSBPL. P1 is an indicator for screening and inhibition of interfering and irrelevant auditory stimuli^74^. A reduction in the P1 amplitude implies a lower demand for auditory processing. SSBPL involves the reading of brand information, such that viewers can focus on processing the auditory information alone. In contrast, MSBPL involves the processing of both music and brand information, which increases the demands of inhibiting auditory interference. The present study demonstrated that the N1 amplitude of SSBPL was larger than that of MSBPL. N1 is an indicator of pitch transitions in the early auditory cortical response^75^. The amplitude of MSBPL is larger under auditory stimulation alone than under joint visual and auditory stimulation^12^. The P2 amplitude of MSBPL was larger than that of SSBPL. Bilateral P2 enhancement reflects the acoustic discrimination of pure tones^76^. Conflicts between auditory speech and visual stimuli will evoke N270^77^. Our results indicated that the N270 amplitude of SSBPL was larger than that of MSBPL. Viewers may have felt a sense of abruptness towards brand information that was irrelevant to the content of the singing program and the program host’s presentation. P3 has been used to detect the brain’s perception of rhythm^78,79^. Moreover, P3 is evoked when capturing rare targets and refocusing attention^77,80^. In the present study, the P3 amplitude of MSBPL was larger than that of SSBPL. For MSBPL, the viewers may have had to divert their attention away from the rhythm of the songs to capture brand information, which in turn shifted and refocused their attention between the two types of information.

The ERSP results indicated that different representations and sounds of brand placement could lead to differences in temporal and spectral EEG dynamics in BA 2, BA 17, and BA 30 (see Section 3.3). Table 2 outlines the statistical results and provides a detailed description.

**Table 2:** Brain region differences for different representations and sounds.

Area	Frequency		Representation	Sound
BA2 (40,−28,33) Right Parietal	γ (>30)	High (>60)	NRE > RE*	—
		Low (30–60)	—	MS > SS*
	β (13–30)		—	MS > SS*
	α (8–13)		RE > NRE*	—
	θ (4–8)		—	—
BA17 (25,−76,10) Right Occipital	γ (>30)	High (>60)	NRE > RE*	MS > SS*
		Low (30–60)	RE > NRE*	—
	β (13–30)		RE > NRE*	MS > SS*
	α (8–13)		RE > NRE*	MS > SS*
	θ (4–8)		NRE > RE*	—
BA30 (−28,−71,9) Limbic Lobe (Left)	γ (>30)	High (>60)	NRE > RE*	MS > SS*
		Low (30–60)	RE > NRE*	MS > SS*
	β (13–30)		RE > NRE*	MS > SS*
	α (8–13)		RE > NRE*	MS > SS*
	θ (4–8)		NRE > RE*	—

Parametric testing with false-discovery rate (FDR) correction was performed for significance testing (*p < 0.05); RE: representable, NRE: non- representable, MS: music sound, SS: speech sound.

A more representable brand placement had a greater impact on the viewers’ brain EEG activity in the right parietal (BA 2) area. Table 2 shows that the different representations of brand placement led to differences in the alpha band of BA 2; REBPL was significantly higher than NREBPL. The natural occurrence of REBPL in real space led to significantly higher spectral power than NREBPL, which involves embedding the graphic logo into the frame after film production and is unrelated to the video script. BA 2, in the parietal region, is responsible for the integration of visual, auditory, and somatosensory information^81,82^, while the parietal alpha band reflects the attentional demand of objects^83^. The PPC plays a role in the integration and processing of spatial discrimination^1,84–87^. Patients with right PPC damage will make errors in tasks involving discrimination of spatial depth^84^, which indicates that the right PPC fulfils the function of spatial discrimination. Activation of the alpha band in the frontoparietal and parietooccipital areas has been used to detect spatial discrimination functions^88,89^. Moreover, high gamma (>60 Hz) in BA 2 was significantly higher for NREBPL than for REBPL. Saccades towards the target location will activate the parietal high-gamma band, while alpha will simultaneously be reduced to suppress the memorised stimulus locations during saccades^90^.

The present study demonstrated that brand placement accompanied by music had a greater impact on the viewers’ brain EEG activity. Different sounds of brand placement led to differences in beta and low gamma (30–60 Hz) in BA 1 and was significantly higher for MSBPL than for SSBPL. BA 2 is the primary somatosensory cortex. Discrimination of auditory stimuli will also activate the somatosensory and motor cortices^26,91,92^. A beta increase was observed when a tone could be imagined to be beat^93^, while discrimination of speech pronunciation activated the left somatosensory cortex^26,94^. Several studies have demonstrated hemispheric differences in the analysis of speech and musical sounds by the brain^26,94^. The right PPC could be involved in the extraction of spatial, pitch, and temporal information from sound signals. It is also related to the right temporal function of retrieving music^95^. When discriminating non-speech sounds, the gamma activation of the right hemisphere is higher than that of the left hemisphere^96^. The frequency for speech discrimination will activate the temporal processing function of the left hemisphere^35,97,98^, while discriminating the melody and tone of music will activate the pitch processing function of the right hemisphere^99,100^. MSBPL involves music and songs composed of melodies and tones with pitch changes and has a regular rhythm. SSBPL is composed of speech sounds without pitch changes, melody, or tone, and does not have a regular rhythm.

The present study revealed that more representable brand placement had a greater impact on the viewers’ brain EEG activity in the right occipital (BA 17) area. Different sounds related to brand placement led to differences in the theta, alpha, beta, and low gamma (30–60 Hz) of BA 17, with those for REBPL being significantly higher than those for NREBPL. BA 17 is the primary visual cortex. Viewing three-dimensional images can better evoke a response in BA 17 than graphic images^101^. Occipital alpha changes can be considered a signal for the allocation of spatial attention^88,102^. In humans, beta activation in BA 17 indicates the processing of pictorial information^103^. Individuals with a greater ability to process pictorial information will evoke higher beta in BA 17 when memorizing pictorial images than those with poorer ability to process pictorial information. REBPL involves the appearance of brand logos in real space and the random presentation of the brand with relevant video content. When viewers view the video content, they will naturally notice the brand logo at the same time. NREBPL involves embedding the brand logo in the same position on the video frame after graphic processing. It is not related to the video content and is present throughout the video. The viewers are almost forced to view the brand logo when viewing the video. Compared with a stationary target, the spectral power of 35–51 Hz (low gamma) will increase when attending to moving targets^104^. In experiments on commercial advertisements^105^, when participants focus their attention on screens with movement or scene changes, there is a drastic decrease in their occipital alpha^22^. Alpha and theta perturbations in the visual cortex also participate in working memory^106,107^. Furthermore, theta and high gamma (>60 Hz) in BA 17 for NREBPL are significantly higher than those for REBPL. Occipital high gamma is related to early visual processing and object discrimination decision making^108,109^. Images that are easily discriminated elicit higher levels of high gamma than those that are not. When selectively attending to visual stimuli, 35–90 Hz (gamma) of the occipital visual cortex will increase^22^.

The present findings demonstrated that brand placement accompanied by music had a greater impact on the brain EEG activity of viewers. Different sounds of brand placement led to differences in alpha, beta, and high gamma in the BA 17, those evoked during MSBPL were significantly higher than those evoked during SSBPL. During MSBPL, brand logos are placed together with musical sounds, involving melodies and tones, whereas during SSBPL brand logos are placed with rhythmic speech that does not involve melodies and tones. Auditory stimulation suppressed the responses of the visual cortex BA 17^94,110^. Auditory stimulation can also alter the subjective (non-physical) perception of the primary visual cortex^111^. When listening to music, alpha, which is a signal for relaxation and introspection, is enhanced^112^. Stimuli with a regular and intense rhythm will evoke a larger beta, while a reduction in the alpha in the right hemisphere reflects linguistic rhythm processing^113^. The induction of beta and gamma can reflect the perceived intensity of tones^93,114^.

Moreover, the present study demonstrated that a more representable brand placement had a greater impact on the viewers’ EEG activity. Different sounds related to brand placement led to differences in the alpha, beta, and low gamma (30–60 Hz) of BA 30, with those evoked during REBPL being significantly higher than those evoked during NREBPL. REBPL involves the presentation of the brand logo in a three-dimensional (3-D) space of actual scenes in the program. Thus, viewers will naturally notice the brand logo. NREBPL, however, involves the graphic processing of the brand logo and embedding it in the same position in the video frame. The logo is unrelated to the video content and is present throughout the video. This may cause viewers to perceive the incongruence of the brand logo, which is unrelated to the video content. BA 30, which is also known as the retrosplenial cortex, participates in contextual analysis^115^. Viewing a 3-D scene leads to greater activation of the BA 30 than viewing close-up scenes, individual objects, and images of human faces^116^. The retrosplenial cortex is the region responsible for analysing 3D geometric spatial information. This region is activated when viewing emotionally stimulating videos or performing episodic memory task, with the left side being related to general memory work, while the right side is related to spatial memory^117^. A decrease in alpha in the retrosplenial cortex not only reflects the demands of visual attention but is also related to coding and retrieval of spatial information^118^., The beta of the retrosplenial cortex will decrease when performing obvious perception or action^119^. Furthermore, the theta and high gamma (>60 Hz) in BA 30 for NREBPL were significantly than those for REBPL. Theta adjacent to the parietooccipital area represents spatial retrieval and decision making^118^, whereas a decrease in theta adjacent to the retrosplenial cortex of the medial temporal lobe reflects spatial processing and memory formation^120^. During short-term spatial memory tasks incongruity between the actual and expected stimuli elicits the synchronicity of theta and high gamma (50–70 Hz)^121^.Another finding from the current study was that brand placement accompanied by music had a greater impact on the viewers’ brain EEG activity. Different sounds of brand placement led to differences in alpha, beta, low gamma, and high gamma in BA 30, with those evoked during MSBPL being significantly higher than those evoked during SSBPL. BA 30 is part of the posterior cingulate cortex and is adjacent to the primary visual cortex^122,123^. Gamma increase adjacent to the left temporal region represents the perception of melodies^124^. Listening and experiencing pleasure from music activates the retrosplenial cortex^125^ and enhances the alpha wave in the brain^112^. Furthermore, listening to musical hallucinations activates beta in the retrosplenial cortex. When actual music has been present for a period, musical hallucinations will become a permanent landmark in the auditory scene, as though the retrosplenial cortex has a positioning effect in the 3D space^124^. Moreover, the gamma of the retrosplenial cortex will increase at rest^126^. MSBPL is a musical performance where a melody accompanies the brand logo information, whereas in SSBPL, brand information is not accompanied by a melody and is only a sound performance.

In summary, the present findings, which were enriched by EEG to record brain electrical activity from the scalp corresponded with the ERSP analysis of differences in the mean spontaneous EEG frequency spectrum under various stimuli conditions and with ERPs measures of averages of specifically time-locked brain responses evoked by different experimental stimuli or events.

The present findings verified the hypothesis that a more representable brand placement will have a greater impact on viewers’ temporal and spectral EEG dynamics. The ERP analysis results demonstrated the significant differences in the P1, N1, P2, N270, P3, and LPC. REBPL induced greater brain responses to spatial processing, emotional stimulation, and attention to rare objects, whereas NREBPL evoked greater responses to stimulus discrimination, conflict processing, and susceptibility to repeated exposures. The ERSP analysis results indicated significant differences in theta, alpha, beta, and gamma (30–100 Hz), which occurred in the right parietal area, right occipital area, and limbic lobe. REBPL also activated the spatial discrimination function of the right parietal area (BA 2); spatial attentional demands, image processing, and tracking of moving targets in the primary visual cortex (BA 17); and the analysis, coding, and memory functions for contextual space in the retrosplenial area (BA 30). NREBPL activated the prosaccadic function of the right parietal area; object judgment and working memory function of the primary visual cortex; and clear perception and processing of incongruent stimuli in the retrosplenial cortex.

The present study verified the hypothesis that brand placement accompanied by music will have a greater impact on viewers’ temporal and spectral EEG dynamics for the discrimination of preferences. The ERP analyses results indicated significant differences in P1, N1, P2, N270, and P3. MSBPL evoked greater brain responses to the suppression of irrelevant auditory information, discrimination of sound rhythms, and capture of rare targets. SSBPL led to greater processing of auditory stimulation alone, and conflicts in auditory and visual information. The ERSP analysis results indicated significant differences in alpha, beta, and gamma (30–100 Hz), which occurred in the right parietal area, right occipital area, and limbic lobe. MSBPL activated the beat imagination and non-speech discrimination functions of the right parietal area (BA 2); perception of tone intensity and regular rhythm in the right occipital region (BA 17); and perception of musical melodies and auditory positioning function of the retrosplenial cortex (BA 30).

## Methods

### Participants

Twenty-five participants (13 men and 12 women), with a mean age of 23.72 years, were recruited for the present study. The participants had university degrees or higher; were residents of Taipei, Taiwan; had corrected visual acuities of ≥0.8; and had no colour blindness, visual impairments, or a history of neurological or psychiatric disorders. The participants did not have a drug or alcohol addiction and were requested to avoid the intake of stimulants (e.g., coffee, alcohol etc.). The study was conducted in accordance with the Declaration of Helsinki^127^. The study was approved by the human trial institutional review board of Cathay General Hospital. All methods were performed in accordance with the approved guidelines. Informed consent was obtained from all participants prior to the experiments.

### Stimuli

Experimental stimuli were obtained from 17 sponsorship singing programs between 2011 and 2014 in China. The 17 Chinese sponsorship singing programs were as follows: ‘The Voice of China’, ‘Sing My Song’, ‘Super Boy’, ‘Super Girl’, ‘Chinese Idol’, ‘Cpop Star’, ‘The X Factor’, ‘Duets’, ‘I am a Singer’, ‘China Sound Super’, ‘The Sing-Off’, ‘China’s New Generation of Sound’, ‘Superstar China’, ‘TCL Perfect Voice’, ‘Blossoming Flowers’, and ‘Bring ‘Em Back’. A total of 48 video clips, which contained features of the independent variables, were selected for the study. The duration of each clip, from which 105 experimental stimuli were extracted, was 25 s. REBPL and NREBLP (or SSBPL and MSBPL) had 105 stimuli. A 2 × 2 Latin square design was used to form the conditional framework for the experimental stimuli, based on the criteria that one factor will occur for stimuli conditions of each row or column^128,129^. The advantages of this design included the counterbalance of the same factor, small error, and high efficiency. Hence, it is frequently applied in quantitative marketing experiments^128–130^. The stimuli conditions of this experiment were as follows: (1) ‘representable versus speech sound’ brand placement, (2) ‘non-representable versus speech sound’ brand placement, (3) ‘representable versus musical sound’ brand placement, and (4) ‘non-representable versus musical sound’ brand placement (Fig. 1A). The resolution of the computer screen presenting the stimuli was 480 × 960 pixels. All programs had not been broadcasted in Taiwan. The presenters and the brands placed in the program had never been seen in the Taiwanese market. Hence, the participants were equally unfamiliar with all stimuli.

### Tasks and Procedures

During the experiment, noise, temperature, light, and other interferences were strictly controlled. The participants provided their responses alone in the EEG laboratory. The researcher observed the participant’s condition and EEG recording from a screen outside the room. A Neuroscan EEG recording and analysis system (Scan 4.3.3 & Presentation), which included an electrode cap (Quik-Cap) and amplifier (SynAmps2), was used. The 32-channel EEG signals were recorded according to the International 10–20 system of electrode placement; experimental facilities referred to in previous studies^4,131–134^ were used for brain sources and signals recording.

Participants viewed a screen placed on a 74-cm-high desktop. The centre of the screen was placed within 10–20° of the participant’s line of sight, at 60–70 cm. The experimental procedure is shown in Fig. 1B. Instructions were given before the experiment, requesting participants to relax and view the stimulus videos, before responding to the experimental questions using the keyboard. Behavioural questions were concerned with the participant’s perception of brand placement. If the participants detected brand logo information placed in the video, they pressed the down key, with no limit to the number of key presses. A ‘+’ fixation point was presented for 1,000 ms in the next screen, followed by the stimulus video for 25,000 ms. The other two behavioural questions were presented at the end of the video. The participants could control their own timings. This process constituted one cycle of the experimental procedure. Forty-eight stimulus videos were randomly presented in 48 trials. A rest period was allowed after every 12 videos, with the participant deciding the length of the rest period. The entire experimental process took approximately 35 min. The following three behavioural questions were presented: (1) Are you aware of the branded product in the video? (2) Do you prefer this form of brand placement? (3) Are you willing to buy the branded product when you see it in the video? The options for response included four levels, with 1 being the most positive attitude and 4 being the most negative attitude. The questions were designed to examine the consumer’s ‘awareness’, ‘preference’, and ‘purchasing desire’ towards the brand placement.

### Behavioural Data Analyses

The behavioural data for each question were then analysed. ‘Awareness’ was calculated as the number of times the participant responded that they perceived brand logo information divided by the total number of stimuli for that stimulus condition., while ‘preference’ and ‘purchasing decision’ were calculated by summing up the participant’s responses. A strongly positive attitude was awarded 4 points; somewhat positive attitude, 3 points; somewhat negative attitude, 2 points; and strongly negative attitude, 1 point. The two factors of brand placement (representation and sound) were used to form four stimulus conditions as follows: (1) representable and speech sound, (2) non-representable and speech sound, (3) representable and musical sound, and (4) non-representable and musical sound. A one-way analysis of variance^135^ was performed to investigate their impact on the participant’s behaviour and attitude.

### Event-related Potential Analysis

Event-relative potentials (ERPs) are brain activity induced by multiple repeated stimulus events. ERP analyses helped determine temporal resolution, which reflected the information processing of the brain at different time epochs^10^. ERPs were distinguished from the recorded EEG signals using superimposition and averaging. ERP data were analysed using the Neuroscan software. Raw EEG data from the 25 participants were first filtered using a band-pass filter. The frequencies of the high-pass and low-pass filters were 0.1 and 30 Hz, respectively. Abnormal segments were deleted; DC signals were corrected to remove electro-oculography interference. EEG waveforms 200 ms before each stimulus and 1000 ms after each stimulus were sliced into epochs and subjected to baseline correction. The time epochs of P1 (90–150 ms)^8,48^, N1 (125–175 ms)^49–51^, P2 (150–250 ms)^13,51,52^, N270 (242–300 ms)^53^, P3 (300–500 ms)^16,54^, and LPC (500–900 ms) were observed. The epochs were then superimposed and averaged to obtain ERPs of brand placement under different conditions.

The overall exclusion rate of ERP epochs for the 25 participants and all stimulus conditions was 9.4%. The exclusion rates for individual stimulus conditions were as follows: ‘representable versus speech sound’, 7.0%; ‘non-representable versus speech sound’, 12.7%; ‘representable versus musical sound’, 9.5%; and ‘non-representable versus musical sound’, 11.6%. Each level of single independent variables has at least 30 epochs for each participant.

Electroencephalography is classically considered as being an excellent temporal resolution among the different brain imaging techniques^1^. Analysis of ERPs, using the average evoked response method, has now become predominant for human experiments over recent decades. The limitation of ERPs was time-locked and phase-locked EEG activity using simple averaging technique enabled to reflect a specific cognitive processing with time-related voltage fluctuations^136^. ERP averaging measures depended on time-locked, more specific response epoch and phase synchronization to brain activity of the event, not only the less phase resolution of EEG, but less overall measures of the time course regarding the underlying sources at scalp level^137,138^. Thus it can be enriched by EEG to record brain electrical activity from the scalp corresponded with the ERSP analysis of differences in the mean spontaneous EEG frequency spectrum under various stimuli conditions.

### Independent Component Analysis and Clustering

By using the algorithms for independent component analysis (ICA) in the EEGLab toolbar^139^, the present study controlled the noise generated during the experimental process and investigated the activation response of each brain region to different methods of brand placement. The underlying assumption of ICA was that the EEG signals recorded from the electrodes were composites originating from neural signals of different brain cortical regions^140,141^. Hence, ICA effectively removed the effects of participants’ eye movements, noise, and electromyographic responses on EEG signals^142,143^. First, the frequency of raw EEG signals for all participants was reduced to 250 Hz, and then a 100-Hz low-pass filter and a 0.5-Hz high-pass filter were applied to reduce sampling and remove noise. EEG data were then segmented into epochs based on the 4 stimulus conditions. Data from 1,000 ms pre-stimulus to 4,000 ms post-stimulus were used for superimposition and averaging. Then, the filtered EEG signals were subjected to ICA to isolate 30 independent components (ICs).

Our study collected a total of 750 ICs (25 participants * 30 ICs) of EEG signals to implement the analysis of ICAs. All EEG signal data computed by the ICAs were characterised by Brodmann brain area used the *K*-means^139,144^ to cluster 750 ICs into the following 12 brain areas: left frontal area, frontal midline area, right frontal area, left temporal area, central area, right temporal area, left parietal area, parietal area, right parietal area, left occipital area, occipital area, and right occipital area. The Talairach *xyz* coordinated with the regions centroids^145,146^ were matched to the Brodmann areas^147^ to investigate their brain functions. However, as shown in Fig. 4, the numbers in each cluster area tended to vary. The weight of ICs shown in the scalp map represented the regional locations of active sources in brain activity, enabling the examination of the source of brain activation in the participants^139^. Subsequently *K*-means clustering was also used in the study of dipole locations and power spectrum density^139,144^. Significant differences of frequency bands oscillations in the ERSP corresponded to the significant brain areas.

### ERSP analysis

In the present study, the ERSP was the spectral perturbation map of the brain, with time as the horizontal axis and frequency as the vertical axis^148^. The EEGLab software was used for ERSP analysis. Wavelet transform was performed to obtain 3- to 100-Hz band intervals to transform the signals of each trial into temporal and frequency data. The Infomax ICA algorithm^141,149^ from the EEGLab toolbox^139^ was employed to effectively find representations for image and audio for tasks such as feature compression and noise removal (e.g. eye movement and blinking, single-electrode noise, muscle activities) when processing EEG signals^142,143,150,151^. Accordingly, the filtered EEG signals with representations were classified into images of independent components corresponding to brain regions. ERSP maps were obtained after the data were normalised using the spectral intensity at baseline to observe intensity changes of the delta (1–3 Hz), theta (4–7 Hz), alpha (8–13 Hz), beta (13–30 Hz), low-gamma (31–60 Hz), and high-gamma (>60 Hz) frequency bands, as well as their corresponding neurophysiological and cognitive implications. Significant between-group differences were analysed using a *T-*test to examine EEG changes in different brain regions when participants viewed the 4 types of stimulus conditions. *P-*values were adjusted using the false discovery rate (FDR)-controlling multiple testing procedure (the fdr.m routine from the EEGLAB toolbox)^152^.

ERSP analysis of event-related dynamics of the EEG spectrum induced by the moment of the beginning of single stimuli trials, were not averaging phase and time-locked response epochs to the stimuli. Moreover, stimuli, such as auditory stimuli, which were not captured by ERP were omitted^138^ since the responses would have elicited a wide range of frequency band oscillations in the ERSP. The ERSP revealed features of event-related brain dynamics that were not contained in the ERP average of the same response epochs^137^. The ERSP was an analysis of single response epochs, multi-channel source localization studies that was capable of obtaining event-related power spectrum density in terms of frequency oscillations from EEG data, which could be phase-coherent/incoherent across selected spatially adjacent or non-adjacent cortical areas, plotted in the plane of time (ms, the horizontal axis) by frequency (Hz, the vertical axis)^153^, corresponding to the temporal-spatial resolution. Efforts to observe and characterise phase-incoherent event-related brain dynamics had been an emerging research topic since^154,155^.
